# Improvement in Thermochromic Offset Print UV Stability by Applying PCL Nanocomposite Coatings

**DOI:** 10.3390/polym14071484

**Published:** 2022-04-06

**Authors:** Marina Vukoje, Rahela Kulčar, Katarina Itrić Ivanda, Josip Bota, Tomislav Cigula

**Affiliations:** University of Zagreb Faculty of Graphic Arts, Getaldićeva 2, 10000 Zagreb, Croatia; katarina.itric.ivanda@grf.unizg.hr (K.I.I.); josip.bota@grf.unizg.hr (J.B.); tomislav.cigula@grf.unizg.hr (T.C.)

**Keywords:** thermochromic printing inks, UV stability, polycaprolactone, nanoparticles, TiO_2_, ZnO

## Abstract

Thermochromic (TC) printing inks change their colouration as a response to a change in temperature. This ability renders them attractive for various applications such as smart packaging, security printing, and marketing, but their application is limited due to their low UV stability, i.e., loss of their thermochromic effect when exposed to UV radiation. In order to improve the UV stability of TC prints, one offset TC printing ink was printed and coated with nanomodified polycaprolactone (PCL) coating. The coating was prepared with the incorporation of 1%, 2%, and 3% mass ratios of ZnO and TiO_2_ nanoparticles in the PCL matrix. The prepared nanocomposite coatings were applied onto the TC print and exposed to UV radiation; afterwards, they were characterized by the colour properties of prints, SEM microscopy, FTIR, and fluorescence spectroscopy. SEM microscopy, FTIR, and fluorescence spectroscopy showed higher rates of polymer degradation, and the results of colour stability indicated that 3% TiO_2_ in PCL matrix gave the best UV stability and protection of TC prints.

## 1. Introduction

Printing inks are used on a range of different products from packaging, commercial printing, protected documents, and ceramics to textiles, as a tool for communication, or as a specific design feature. In order to fulfil consumers’ expectations, graphic products should be functional and attractive; in this sense, printing inks can play an important role. Thermochromic (TC) printing inks are just one example of how a graphic product can dynamically interact with consumers. Thermochromic printing inks change because of the influence of temperature, and due to that behaviour, they can convey a message to the consumer on the basis of the ink colour they perceive. Thermochromic (TC) materials are available in two forms: TC materials based on leuco dyes and on liquid crystals. In addition, TC materials may be reversible or irreversible. Reversible leuco-dye-based TC materials consist of at least three components, namely, colourant, colour developer, and solvent, which mutually participate in two competitive reactions causing the thermochromic effect. The first reaction occurs between colourant and developer when the coloured complex is formed (at lower temperatures, when the solvent is in solid state), while the other reaction occurs between solvent and developer (at higher temperatures, when organic solvent with the increase in temperature becomes liquid, consequently causing the breakdown of the colourant–developer complex). These reactions allow for the thermochromic complex with a cooling process to return to its original state [1]. Thermochromic printing inks are a mixture of TC pigments and binders. TC pigments are microencapsulated leuco-dye–developer systems responsible for the creation of the TC effect, and they are usually about 10 times larger than the particle size of conventional ink pigments [2]. Ink binders may be different depending on the printing processes for which they are intended or the preferred application. 

In addition to their functionality and attractiveness, these inks also have some drawbacks that are mainly related to their poor stability to various chemicals and light [3,4]. Their poor stability when exposed to UV radiation limits the time of exposure of products to external conditions and limits their applications [4,5,6]. The polymer envelope is much more stable than the mere polymer binder of thermochromic ink [1]. Colour formers used in thermochromic systems have poor light fastness properties [7]. UV absorbers play a very important role in improving the light fastness of colourants, while zinc and nickel 2,4-dihydroxybenzophenone-3-carboxylates were proposed as effective stabilizers against the fading of colour formers [7,8,9]. Moreover, due to the very complex system of TC inks, the photo-oxidation stability of thermochromic prints is affected by the chemical composition of the binder and the TC microcapsules, interactions between printing ink binder and microcapsules, and the ink binder drying mechanism [10]. The increase in the stability of thermochromic inks to UV radiation was the subject matter of an increasing number of studies. Friškovec et al. showed that a UV protective layer can help in preserving the dynamic colour properties of TC prints [4]. Rožić et al. showed that small proportions (up to 20%) of zeolite tuff in the paper used as a filler act favourably on the UV stability of TC prints, while higher proportions reduce stability as an effect of photocatalytic oxidation features due the presence of Fe oxides in the sample of natural zeolite tuff [5].The stability of TC printing inks may also be influenced by the ink composition [11], i.e., mineral-oil-based TC inks have a lower degree of photostability than that of TC printing inks based on vegetable oil. Rožić and Vukoje showed that microcapsules in UV screen printing ink show higher stability to photo-oxidation compared to microcapsules present in offset print due to the different interactions of TC microcapsules with the ink binder from their chemical composition [10]. Despite the fact that TC printing inks have low UV stability, the overprint UV protection of TC prints was not intensively studied.

As overprint coatings, different materials and techniques can be used, such as lamination and varnishes, but these materials generally have some drawbacks due to environmental concerns. Thus, in the last decade, the use of biodegradable materials has been the subject of numerous studies. Even though they are environmentally friendlier, their lower stability to environmental factors, such as lower stability to photo-oxidation, limits their applications. In order to improve the photostabilisation of polymers, different stabilisation systems can be used as additives; on the basis of stabiliser action, they can be light screeners, UV absorbers, excited-state quenchers, peroxide decomposers, and radical scavengers [12]. Organic materials are used in the manufacture of UV absorbers, but their limited lifetime due to photodegradation, phototoxicity, and photoallergenic effects limits their practical applications. Therefore, considerable efforts were undertaken for the development and use of inorganic materials, such as metal oxides (e.g., ZnO, TiO_2_ and SiO_2_, in the formulation of UV absorbers [13,14,15]. Inorganic UV absorbers (nano-metal oxides) attract much attention in the field of smart packaging materials due to their excellent biocompatibility and functional properties [16,17]. In addition, they are usually less toxic, environmentally friendly, thermally or chemically stable, with better resistance, and a wider range of UV blocking capability. Among them, nano-TiO_2_ is widely used because of its reasonable price, nontoxicity, and light stability, in addition to its good UV blocking function, and the ability to reduce light transmission in UV-A and UV-B light, which plays an important role in the prevention of photo-oxidation [18]. Zinc oxide (ZnO) with high-efficiency UV absorption resulting from a wide band is currently listed as a generally safe (GRAS) material by the U.S. Food and Drug Administration [19]. 

With that in mind, this study investigates the possibility of creating nanocomposite coating from polycaprolactone and metal oxides at the nanoscale, which in other studies showed good behaviour and resistance to different environmental factors when it comes to conventional printing inks [20,21,22,23]. Poly(ε-caprolactone) or polycaprolactone (PCL) is biodegradable synthetic polyester. Recent studies showed the potential of using PCL nanocomposite coating to enhance the surface and mechanical properties, and UV stability of conventional offset prints [20,21,24,25]. Delgado Lima and Botelho found that TiO_2_ nanoparticles enhance polymer chain scission during the UV exposure of PCL/TiO_2_ nanocomposites, while the increased amount of inorganic nanoparticle nanocomposites exhibited lower thermal stability [26]. In addition, França et al., and Tsuji eta al. stated that PCL photodegradation takes place through the bulk erosion mechanism [27,28]. 

Although overprint varnishes are often used to protect prints from mechanical damage, this paper proposes nano-modified PCL coating for the improvement of thermochromic offset print UV stability while preserving its effect. Due to the different formulation and size of colourants, thermochromic offset inks differ from conventional offset inks, which in the end affects their UV stability. In general, conventional offset prints are far more stable in comparison to thermochromic offset prints. Hence, the objective of this study was to investigate the possibility of improving TC print UV stability. So far, there are no data on TC print UV stability protection through coating application. For this research, a typical commercially available TC offset ink based on leuco dyes was used. The used ink can be printed on different printing substrates, but in all cases, the same behaviour can be expected, i.e., the same thermochromic colour effect. In the PCL solution, two nano-metal oxides (TiO_2_ and ZnO) were mixed in 1%, 2%, and 3% mass ratios. In addition to UV protection, this study examines the influence of different wight concentrations of nanoparticles (NPs) in a PCL matrix on the thermochromic colour change effect.

## 2. Materials and Methods

### 2.1. Printing Ink

One commercially available thermochromic offset ink based on leuco dye was printed on commercially available uncoated paper for the purpose of preparing samples for further testing. The activation temperature of thermochromic ink is 45 °C. Below its activation temperature, the print was coloured in green, and above its activation temperature, the print was coloured in yellow. The printing process was carried out using a Prüfbau Multipurpose Printability Tester (Prüfbau Peissenberg, Germany). A quantity of 1.5 cm^3^ ink was applied on the distribution rollers, while printing was carried out with printing force of 600 N. All samples were printed in the full tone (marked as P) under the same conditions. 

### 2.2. Coating Preparation

The coating was prepared by the dissolution of PCL polymer granulates (Sigma Aldrich, St. Louis, MO, USA) in ethyl-acetate solvent (Kemika, Zagreb, Croatia). Using a heated magnetic stirrer, the solution was heated to 40 °C and stirred for about 30 min to obtain a homogenic solution. The coatings on the printed samples were prepared from a PCL polymer solution by varying the concentrations of TiO_2_ and ZnO nanoparticles (NPs) (Table 1). Seven different coatings were prepared: without nanoparticles or neat PCL (P/PCL); by adding 1 mass % (P/PCL/1Ti), 2 mass % (P/PCL/2Ti), and 3 mass % (P/PCL/3Ti) of titanium dioxide (TiO_2_, Aeroxide P25, Evonik industries AG, Essen, Germany); and 1 mass % (P/PCL/1Zn), 2 mass % (P/PCL/2Zn), and 3 mass % (P/PCL/3Zn) of zinc oxide (ZnO, Lach-Ner, Neratovice, Czech Republic), and dispersing them with an IKA T 25 digital Ultra-Disperser (IKA-Werke, Staufen, Germany) for 8 min at 15,000 rpm. Unwanted solvent evaporation was controlled by sealing the container using paraffin strips, and the mass of the solution was compared before and after the procedure.

### 2.3. Coating Application

The coatings were applied using a K202 Control Coater (RK Print, Litlington, UK) in controlled conditions defined by the ISO 187:1990 standard. Wet coating thickness was defined with the standard coating bar to 24 μm (dry coating ~6 μm). All coatings were applied on the printed side of the paper and designated according to Table 1. 

### 2.4. Exposure of Samples to UV Radiation

For the evaluation of UV stability of coated thermochromic prints, samples were exposed to UV radiation in a Solarbox 1500e device (CO.FO.ME.GRA, Milano, Italy), with controlled temperature and UV radiation. All samples were exposed to filtered xenon light for a period of 6, 12, and 18 h at a BST temperature of 40 °C and irradiation of 550 W/m^2^. The indoor UV filter was used to eliminate the UVB spectral range from the xenon spectral distribution curve. The used filter in that form allows for simulating sun rays filtered through a windowpane, i.e., it simulates the conditions of internal exposure. 

### 2.5. Determination of UV–Vis Absorption Spectra of TiO_2_ and ZnO Nanoparticles

For the measurement of the TiO_2_ and ZnO nanoparticles’ UV absorption property, prior to measurement, a small amount of nanoparticle powder was placed between a quartz glass microscope slide and a quartz glass cover slip in order to prevent the powder material from penetrating the spectrophotometer’s sphere aperture. An Ocean Optics USB 2000+ spectrometer (Ocean Optics, Orlando, FL, USA) with a 30 mm wide integrating sphere under (8:di) measuring geometry (diffuse geometry, specular component included) and the addition of deuterium–tungsten halogen UV light source DH-2000 (Ocean insight, Orlando, FL, USA) was placed on the upper side of the quartz glass cover slip; measurements were performed in steps of 1 nm for the spectral region from 200 to 800 nm. 

### 2.6. Colorimetric Measurement

Spectral reflectance was measured by using an Ocean Optics USB2000+ spectrometer (Ocean Optics, Orlando, FL, USA) with 30 mm wide integrating sphere under (8:di) measuring geometry (diffuse geometry, specular component included). Printed samples (uncoated and coated) were heated–cooled on a full-cover water block (EK Water Blocks, EKWB d.o.o., Komenda, Slovenia) whose temperature was varied with a thermostatically controlled water block. Reflectance spectra were measured in one heating–cooling cycle by heating samples from 25 to 55 °C and then cooling them back to 25 °C. Measurements were performed in steps of 1 nm for the spectral region from 400 to 800 nm. Ocean Optics SpectraSuite software (version 2.0.8) was used for the calculation of the CIELAB values from the measured reflectance. D50 illuminant and 2° standard observer were applied in these calculations. Colour differences were calculated using the CIEDE2000 total colour difference formula [29]. 

### 2.7. SEM Microscopy

Uncoated and coated thermochromic (TC) prints before and after exposure to UV radiation were monitored using a Tescan Vega III Easyprobe (Tescan, Brno, Czech Republic) field-emission scanning electron microscope. Micrographs were taken under magnification of 5000×.

### 2.8. FTIR Spectroscopy

ATR spectra of the samples before and after UV exposure were measured using a Shimadzu FTIR IRAffinity-21 spectrometer (Shimadzu, Kyoto, Japan) with Specac Silver Gate Evolution as a single-reflection ATR sampling accessory with a ZnSe flat crystal plate (index of refraction, 2.4). IR spectra were recorded in the spectral range between 4000 and 400 cm^−1^ at 4 cm^−1^ resolution and averaged over 15 scans. 

### 2.9. Fluorecence Spectroscopy

Fluorescence intensity was measured using an Ocean Optics USB4000+ spectrometer 2000 (Ocean Optics, Orlando, FL, USA) in combination with a 30 mm wide integrating sphere under (8:di) measuring geometry (diffuse geometry, specular component included) with the addition of an LSM Series LED 2000 (Ocean insight, Orlando, FL, USA) light source at 365 nm as the fluorescence excitation wavelength. The use of an integrating sphere in fluorescence measurements were previously discussed and applied in different areas [30,31,32,33]. The excitation light source was kept stable at a constant current of 0.140 A. Fluorescence intensity was measured in the spectral range between 330 and 630 nm.

## 3. Results and Discussion

### 3.1. UV–Vis Absorption Spectra of TiO_2_ and ZnO Nanoparticles

**Figure 1:**
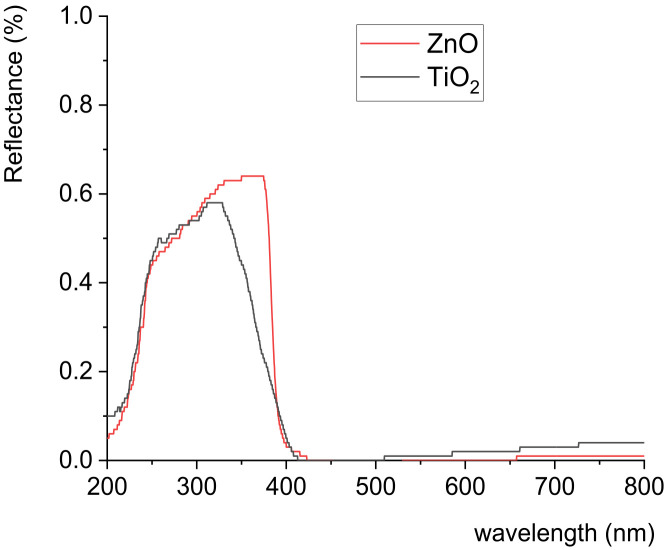
UV–vis absorption spectra of ZnO and TiO_2_ nanoparticles.

UV–vis absorption spectra of the used nanoparticles (ZnO and TiO_2_) are presented in Figure 1. The band gap of ZnO is similar to that of TiO_2_, but the ZnO band gap is somewhat larger. Figure 1 shows that ZnO nanoparticles absorbed radiation in a UV range up to 374 nm, and almost all visible spectral radiation was scattered by the ZnO nanoparticles, while TiO_2_ nanoparticles absorbed UV irradiation up to 329 nm. Obtained results are similar to those observed in another study [34]. UV absorption edges of the used nanoparticles differed in the case of TiO_2_ being shifted towards lower wavelengths. The UV absorption edge can be influenced by the size of the nanoparticles [34]. 

### 3.2. Colorimetric Properties of Uncoated and Coated TC Prints

**Figure 2:**
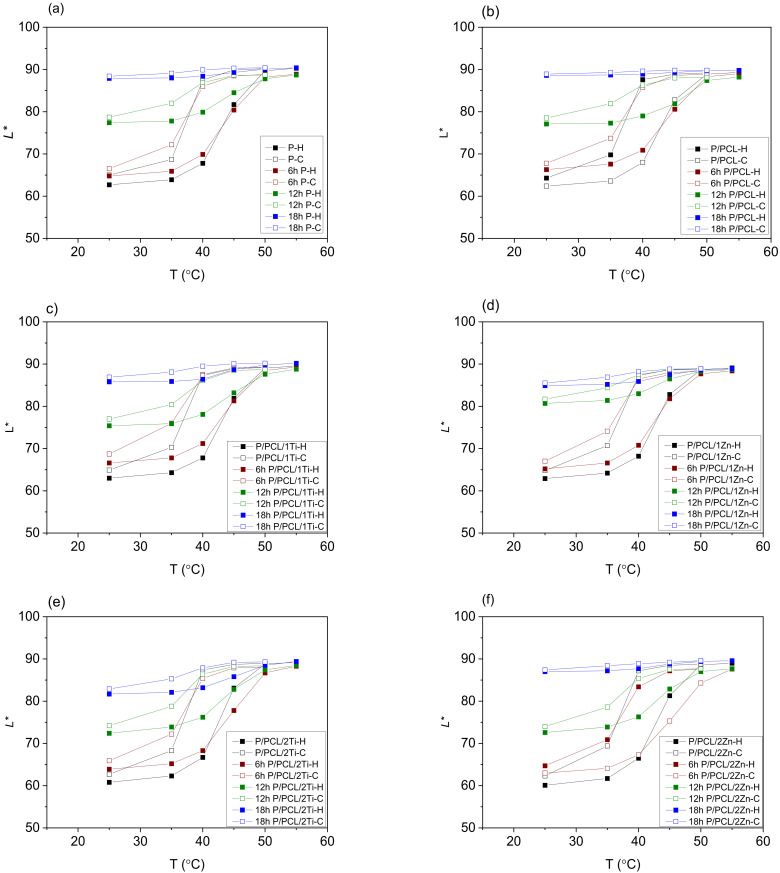

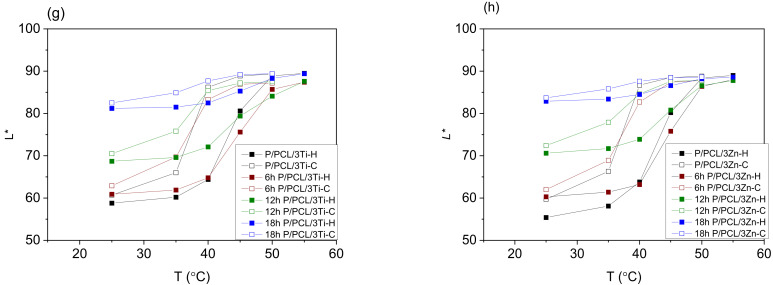
Colour hysteresis of (**a**) uncoated TC print, (**b**) PCL coated TC print, (**c**) PCL/1%Ti coated TC print, (**d**) PCL/1% Zn coated TC print, (**e**) PCL/2% Ti coated TC print, (**f**) PCL/2% Zn coated TC print, (**g**) PCL/3% Ti coated TC print, (**h**) PCL/1% Zn coated TC print; before and after exposure to UV radiation for 6, 12, and 18 h.

To determine the influence of coating application and UV radiation on the thermochromic effect (the dynamic colour change) of TC prints, prepared samples were analysed through the colour hysteresis, spectral reflectance curves, and total colour difference (TCD) of a sample measured between heating and cooling at a temperature well below the final chromic temperature (Figure 2 and Figure S1). Colour hysteresis (Figure 2 and Figure S1) describes the temperature dependence of prints for the L* component of colour. Colour hysteresis is commonly called thermochromic memory [1]. The process is illustrated by the change in lightness L* as a function of temperature. The CIELAB values of thermochromic print samples were calculated by applying measured reflectance spectra during heating from 25 to 55 °C and cooling down to 25 °C. At a temperature lower than the activation temperature (45 °C), microcapsules are blue, while they become yellow above that temperature. 

The application of PCL nanocomposite coating caused notable differences in colour hysteresis, for example, for samples coated with nanomodified PCL coating, the lightness value at lower temperatures became smaller, i.e., the initial colouration of the samples appeared to be darker (Figure 2 and Figure S1). Dailliez et al. showed that, in the case of halftone prints, the application of a smooth transparent layer darkens the print and saturates its colour due to the lateral propagation of light within the coating during the multiple-reflection process that occurs between the printed diffusing substrate and the coating–air interface [35]. In this case, changes in colour can also be related to the presence of nanoparticles. With the increase in NP ratio in the PCL matrix, samples became darker at lower temperatures. This behaviour was observed for all samples containing ZnO and TiO_2_ nanoparticles, with somewhat greater changes (lower L*) for ZnO nanomodified coating. This behaviour can be explained by the increase in the number of NPs in the coating, i.e., the increase in the amount of absorbed light and consequent lower reflection. In the case of ZnO NPs, the higher band gap (Figure 1) caused a higher amount of absorbed light resulting in the darker appearance of samples in comparison to TiO_2_, which had a smaller band gap and absorbed less light, resulting in greater light reflection. 

Figure 2 shows the influence of UV radiation on the TC prints’ colour hysteresis loops. TC memory gradually degrades with the increase in exposure time to UV radiation. Their height becomes smaller (the result of reduced TCD), and slopes decrease a little more when cooled (denoted as -C) and less when heated (denoted as -H). After 6 h of exposure to UV radiation, all hysteresis loops were modified. Moreover, 18 h of UV exposure produced remarkable changes in the TC effect, which was completely destroyed for the uncoated TC print (Figure 2a) and the print coated with neat PCL (Figure 2b); therefore, there were no dynamic colour properties, whereas the resulting loop remained very small on TC prints coated with nanomodified PCL with 1% TiO_2_ (Figure 2c), 1% ZnO (Figure 2d), and 2% ZnO (Figure 2f). Higher nanoparticle concentrations resulted in better protection, but the 3% TiO_2_ content (Figure 2g) stood out on TC samples, as it offered better protection than that of 3% ZnO (Figure 2h). Best results were obtained for TiO_2_-coated samples, where these loops were wider, resulting in a more visible colour change effect.

The thermochromic effect increasingly decreased with the exposure of samples to UV radiation, and the reversible colour change was increasingly less present. The range and dynamics of colour through the heating and cooling cycle were reduced, which was especially evident in samples exposed to 18 h of UV radiation. On PCL-treated samples, the TC effect completely disappeared after 18 h of exposure to UV radiation, while the highest colour change dynamics was visible on TC prints coated with PCL modified with 3% TiO_2_ coating. 

**Figure 3:**
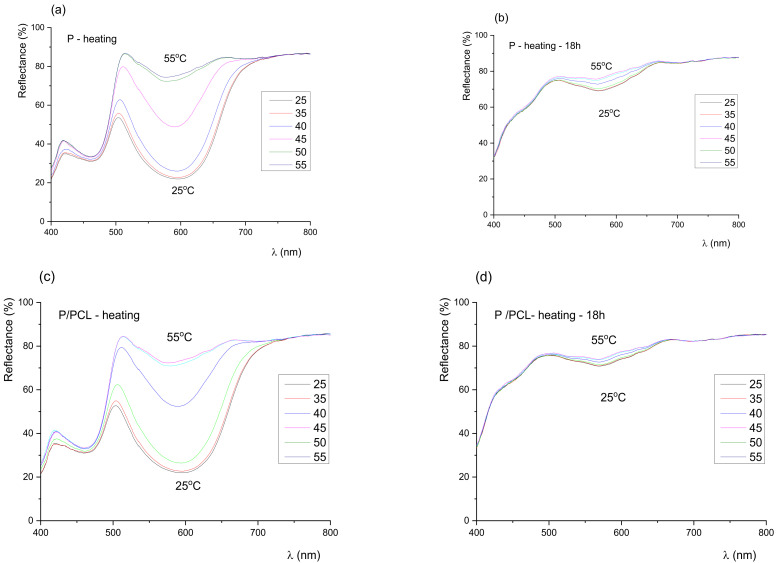
Reflectance spectra during heating process of (**a**) uncoated TC print (untreated), (**b**) uncoated TC print exposed to UV radiation for 18 h, (**c**) PCL coated TC print (untreated), (**d**) PCL coated TC print exposed to UV radiation for 18 h.

**Figure 4:**
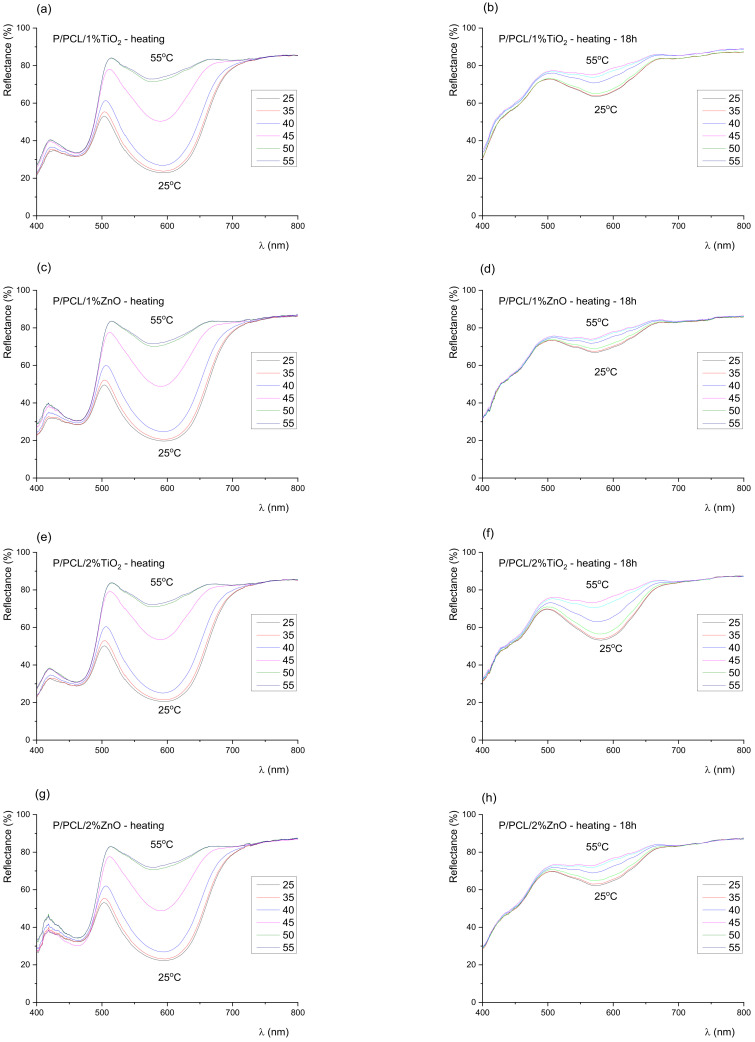

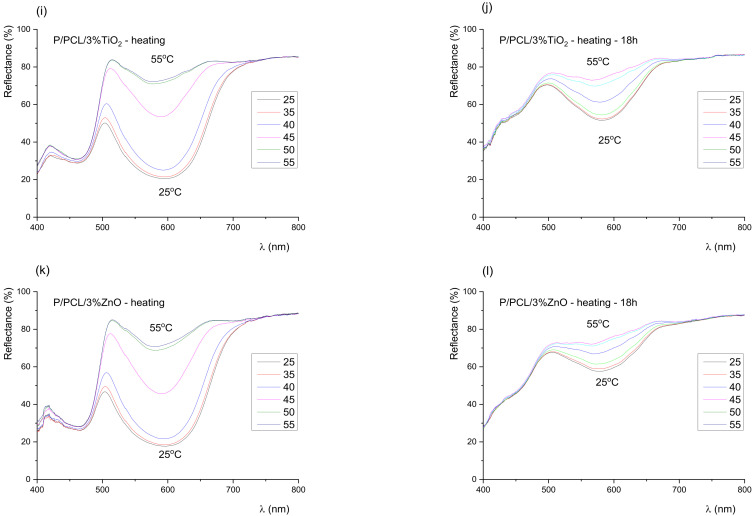
Reflectance spectra of TC prints measured during heating, coated with (**a**) PCL/1% Ti (untreated), (**b**) PCL/1% Ti after 18 h of exposure to UV radiation, (**c**) PCL/1% Zn (untreated), (**d**) PCL/1% Zn after 18 h of exposure to UV radiation, (**e**) PCL/2% Ti (untreated), (**f**) PCL/2% Ti after 18 h of exposure to UV radiation, (**g**) PCL/2% Zn (untreated), (**h**) PCL/2% Zn after 18 h of exposure to UV radiation, (**i**) PCL/3% Ti (untreated), (**j**) PCL/3% Ti after 18 h of exposure to UV radiation, (**k**) PCL/3% Zn (untreated), (**l**) PCL/3% Zn after 18 h of exposure to UV radiation.

Results of spectral reflection curves (Figure 3 and Figure 4) show that the colour of the samples before exposure to UV radiation remained green until the activation temperature (45 °C), after which it slowly turned yellow. No sudden changes were observed, and the colour of the prints continuously changed with the increase in temperature. With exposure of the samples to UV radiation, the dynamics of colour change on the sample rapidly changed. Results of the spectral reflection curves show that with exposure of the samples to UV radiation after 18 h, part of the 400–450 nm spectrum was gradually lost, which indicated the degradation of the blue TC pigment, i.e., the degradation of the microcapsules. In the green (500–570 nm) and yellow–orange spectral range (570–630 nm), even after 18 h of exposure to UV radiation, a change was visible. Changes in the green area indicated that not all blue microcapsules were damaged by UV radiation, unlike the uncoated print and print coated with neat PCL coating, where the thermochromic effect had almost disappeared.

**Figure 5:**
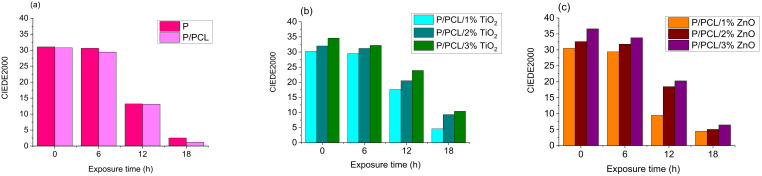
Total colour difference (TCD) between two states (measured at 25 and 50 °C) for (**a**) unprinted TC print and TC print coated with neat PCL, (**b**) TC print coated with 1, 2, 3% TiO_2_ nanomodifed PCL, (**c**) TC print coated with 1, 2, 3% ZnO nanomodifed PCL, before and after 6, 12, and 18 h exposure to UV radiation.

A TC sample is considered to work properly if the total colour difference between the two states below and above the activation temperature is well-visible. The total colour difference (TCD) between the two states for samples unexposed and exposed to UV radiation is shown in Figure 5, which indicates that the application of PCL coating over the TC print increased the total colour difference of the original samples with the increase in NP share in the formulation of PCL coating. This behaviour was also visible on the obtained hysteresis (Figure S1). TCD gradually decreased with the increased time of UV exposure.

Reversible colour change was present in all samples, implying that the thermochromic effect had not been lost during UV degradation, but it was significantly reduced after 18 h of UV irradiation. After 18 h of UV exposure, the colour change effect completely disappeared in the PCL sample and fell below 3 CIED2000 units, even more than that for the untreated sample. Figure 5 shows that nanoparticle deposition is effective, as the difference in TC effect between treated and untreated samples was quite obvious. Best results were obtained for the 3% TiO_2_ nanomodified PCL coating. 

Colorimetric results show that the incorporation of the greatest investigated amount (3%) of TiO_2_ nanoparticles in PCL matrix significantly impacted the UV stability of TC prints. The UV stability of the uncoated TC print and the print coated with neat PCL was lower than that on prints coated with 3% TiO_2_ nanomodified PCL coating, probably because the print under the PCL polymer layer absorbed UV radiation with TiO_2_ nanoparticles, thus preventing UV radiation from penetrating into the print. 

### 3.3. SEM Microscopy

**Figure 6:**
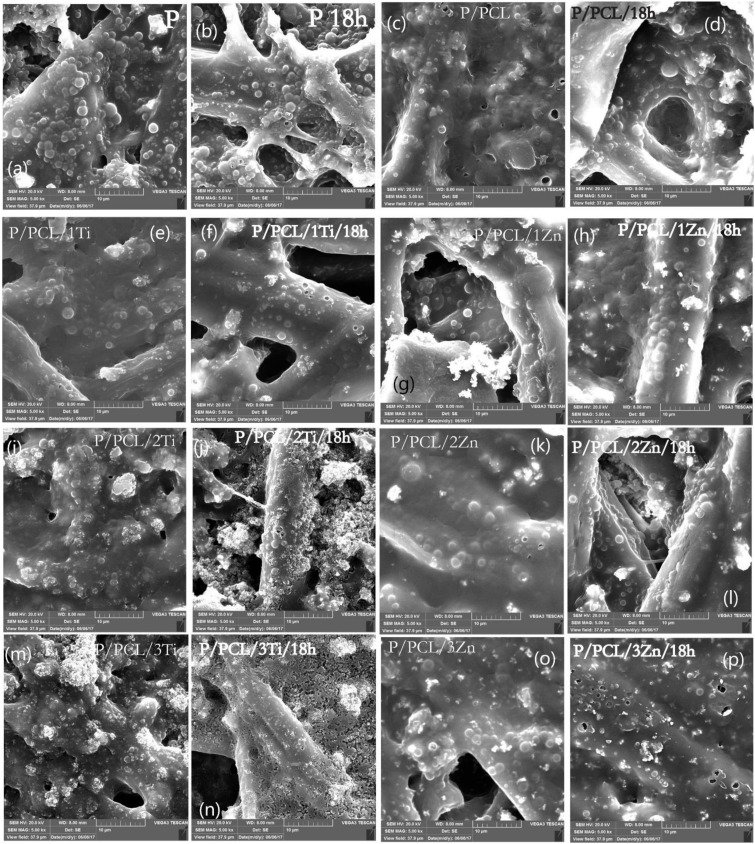
SEM micrographs of (**a**) uncoated TC print, (**b**) uncoated TC print after exposure to UV radiation for 18 h, (**c**) TC print coated with neat PCL, (**d**) TC print coated with neat PCL after exposure to UV radiation for 18 h, and coated TC prints with nanomodified PCL (**e**) PCL/1% Ti (untreated), (**f**) PCL/1% Ti after exposure to UV radiation for 18 h, (**g**) PCL/1% Zn (untreated), (**h**) PCL/1% Zn after exposure to UV radiation for 18 h, (**i**) PCL/2% Ti (untreated), (**j**) PCL/2% Ti after exposure to UV radiation for 18 h, (**k**) PCL/2% Zn (untreated), (**l**) PCL/2% Zn after exposure to UV radiation for 18 h, (**m**) PCL/3% Ti (untreated), (**n**) PCL/3% Ti after exposure to UV radiation for 18 h, (**o**) PCL/3% Zn(untreated), (**p**) PCL/3% Zn after exposure to UV radiation for 18 h.

Figure 6 shows the SEM micrographs of samples before and after exposure to UV radiation for 18 h. SEM micrographs show that the surface of TC print before exposure to UV radiation was heterogeneous, with the presence of significant number of TC microcapsules in regular circular forms with around 1–3 µm diameter and covered with a thin layer of ink binder. In addition, the penetration of microcapsules in the paper structure between the cellulose fibres was visible. SEM micrographs do not show the significant degradation of TC microcapsules after the print had been exposed to UV radiation since only several holes in TC microcapsules are visible. The masking of degraded TC microcapsules probably occurred by the ink binder and their penetration between the cellulose fibres. The formation of craters probably occurred due to degradation of ink binder. 

PCL-coated prints showed that the TC microcapsules were covered with a thicker layer, indicating the presence of a PCL layer over the TC print. As a nanoparticles weight increases in a coating, TC microcapsules are less visible and a number of bright spots occurs, which may be an indication of nanoparticles agglomeration. 

In the case of samples exposed to UV radiation, the greatest changes were visible on samples coated with TiO_2_ nanomodified PCL, namely, 2% and 3%. In all cases, the heterogeneous degradation of the samples occurred with visible surface erosion. Results indicate that the addition of TiO_2_ nanoparticles into the PCL matrix more efficiently promoted UV PCL decomposition with regards to ZnO nanoparticles. All changes were more visible as the amount of TiO_2_ NPs in the PCL matrix increased. In the case of the addition of 3% ZnO into the PCL matrix, holes appeared on the TC microcapsules. No significant change in PCL was observed as in the case of the ZnO nanomodified coating. This may indicate different mechanisms of photodegradation. 

### 3.4. FTIR Spectroscopy

**Figure 7:**
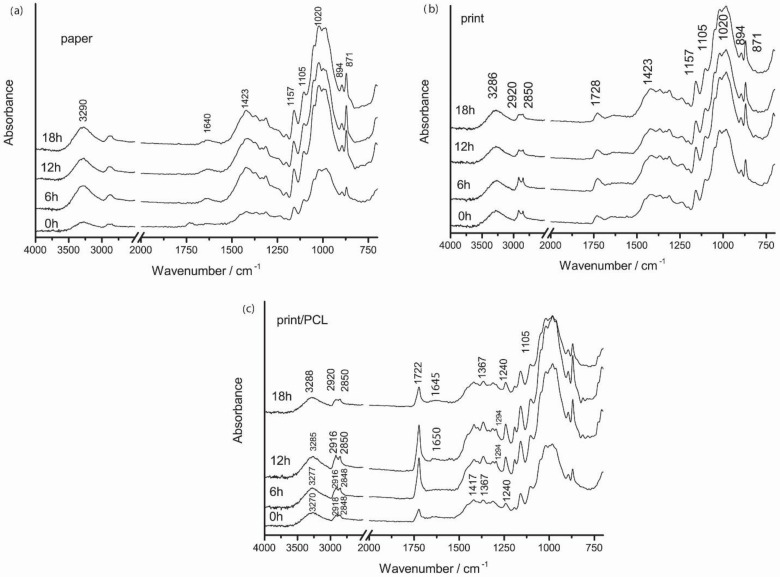
FTIR spectra of (**a**) paper, (**b**) TC print, and (**c**) TC print coated with neat PCL coating before and after 6, 12, and 18 h exposure to UV radiation.

**Figure 8:**
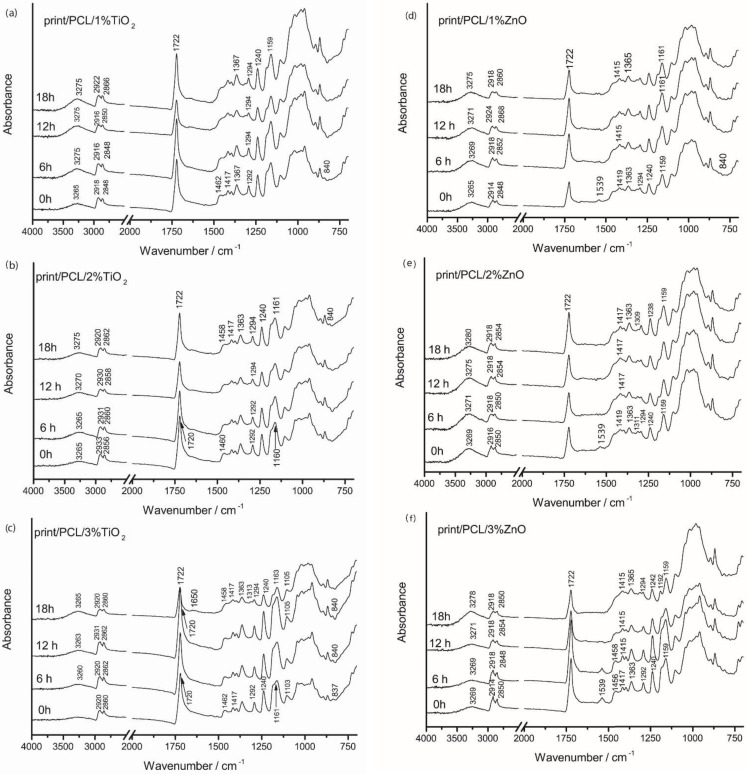
FTIR spectra of TC print coated with nanomodified PCL coatings (**a**) PCL/1% Ti, (**b**) PCL/2% Ti, (**c**) PCL/3% Ti, (**d**) PCL/1% Zn, (**e**) PCL/2% Zn, (**f**) PCL/3% Zn before and after 6, 12, and 18 h exposure to UV radiation.

FTIR spectra of all sample surfaces are presented in Figure 7 and Figure 8. In the FTIR spectra of the used paper, vibrational bands of cellulose (1423, 1157, 894 cm^−1^) calcium carbonate (871 cm^−1^), and adsorbed water (1640 cm^−1^) were visible [36]. The broad band around 3290 cm^−1^ in the paper spectrum was assigned to the –OH stretching of hydroxyl groups in cellulose. Vibrational bands around 2910 and 2850 cm^−1^ were mostly influenced by additives in the papermaking process [37].

In the case of the FTIR spectra of the neat uncoated TC print, vibrational bands of used thermochromic printing ink most likely originated from the thermochromic ink binder, mainly vegetable oils as the common component of the ink binder. Thus, vibrational bands could not have been the result of the vibrational modes of the thermochromic composites within the microcapsules or the TC microcapsules’ polymer shell since they were covered with the ink binder [38,39]. Due to the coverage of microcapsules with the ink binder, the vibrational bands of the microcapsules’ wall material were probably covered and overlapped with the vibrational bands of the ink binder that was present in higher amounts. Regarding the FTIR spectra of thermochromic print, a significant contribution of the cellulose bands could be noticed due to the penetration of the TC printing ink into the paper structure, especially in the 1500–800 cm^−1^ spectral range. The carbonyl stretching band of the thermochromic ink at 1728 cm^−1^ was present in the FTIR spectrum of the TC print, as opposed to the FTIR spectrum of unprinted paper. Bands at 2920 and 2850 cm^−1^ in the spectrum of thermochromic print were assigned to the –CH_2_ and –CH_3_ stretching bonding vibration of aliphatic chains, respectively, and implied the presence of oils in the printing ink binder [40]. 

With the exposure of paper to UV radiation, changes in the FTIR spectra could be observed in the spectral range of 1500–1850 cm^−1^, where carbonyl groups appeared but only if the bending vibrations of the bound water molecules at 1640 cm^−1^ were not present since they could mask the products of cellulose oxidation [41]. In our case, adsorbed water molecules were present and probably masking the vibrational bands of paper degradation products. No additional changes in the IR spectra of unprinted paper during 18 h of UV irradiation were observed, indicating that this relatively short radiation time (18 h) slightly affects paper stability and its properties. 

Characteristic vibrational bands of the PCL polymer were obtained at 2918 and 2848 cm^−1^, attributed to the stretching of aliphatic C–H groups, the ester carbonyl (C=O) stretching absorption band at 1722 cm^–1^, and the asymmetric stretching of C–O–C bonds at 1240 cm^−1^, while their symmetric stretching corresponded to the peak at 1162 cm^−1^ [24,41,42]. With the application of PCL coating over the TC print, the carbonyl peak was shifted to smaller wavelengths at around 1722 cm^−1^, unlike the TC print, where it was located at 1728 cm^−1^. This may indicate overlapping carbonyl groups vibrational bands. 

By the exposure of the uncoated print to UV irradiation, changes in the spectral range of 2920–2850 cm^−1^ occurred. These changes are related to the –CH_2_ and –CH_3_ stretching bonding vibrations of aliphatic chains, which imply the presence of oils in the printing ink binder, which indicates binder degradation. By the exposure of prints coated with PCL to UV irradiation, a decrease in vibrational bands in the spectral range of 2920–2850 cm^−1^, and a shift towards greater wavenumbers occurred. In addition, a decrease in vibrational bands intensities at 1417, 1367, 1294, and 1240 cm^−1^ occurred. The band at 1294 cm^−1^ gradually disappeared after 18 h of exposure to UV radiation.

The application of PCL–TiO_2_ nanomodified coating (Figure 8a–c and Figure S2) resulted in the partial masking of cellulose vibrational bands that were present in the FTIR spectra of the TC print and PCL-coated thermochromic print (Figure 7b,c). The addition of TiO_2_ and ZnO nanoparticles to the PCL matrix lead to changes in the IR spectrum of PCL, i.e., functional groups, their displacement, and the formation of new bands and their shoulders, which indicated a change in the crystal structure of PCL (Figure S2). By the incorporation of TiO_2_ and ZnO nanoparticles into the PCL matrix (Figure S2), the vibrational bond at 1160 cm^−1^ was broadened, and the shoulders at 1186 cm^−1^ in the case of TiO_2_ particles and at 1190 cm^−1^ in the case of ZnO particles were formed. In that spectral range (from 1167 to 1191 cm^−1^), a symmetrical vibration describes C–O bonds. The vibration at 1191 cm^−1^ was attributed to O–C–O bond stretching, while the vibrational bond at 1166 cm^−1^ corresponded to CH_2_ deformation [24,42]. Furthermore, the addition of 1%, 2%, and 3% TiO_2_ nanoparticles (Figure 8a–c) lead to the formation of vibrations bands at 1462 and 1363 cm^−1^, which corresponded to the C–H bond stretching of alkanes, while absorbance at 729 cm^−1^ represents the vibration of –CH_2_ bonds. In addition, through the incorporation of TiO_2_ nanoparticles, the vibrational band at 1293 cm^−1^ was formed that is related to the crystalline phase of PCL (C–C and C–O) [42,43]. The formation of the crystalline band at 1293 cm^−1^ was also noticed in the case of addition 3% TiO_2_ nanoparticles. The vibrational band at 1462 cm^–1^ was attributed to the axial symmetric deformation of CH, while the band at 1292 cm^–1^ could be assigned to backbone C–C (=O)–O, the stretching vibration of the crystalline phase of PCL.

In the case of the nanomodified ZnO PCL coating, differences could be seen in the FTIR spectra (Figure 8d–f and Figure S2). IR spectra indicated a lower degree of PCL crystallinity in comparison to that of TiO_2_ nanomodified coating. Only the addition of 3% ZnO NPs into the PCL matrix resulted in somewhat similar IR spectra to those of the TiO_2_ nanomodified coatings (Figure 8f). Moreover, the addition of ZnO NPs into the PCL matrix caused the formation of the vibrational band at 1539 cm^−1^, which increased with the increase in ZnO NPs amount, which could be attributed to the main ZnO characteristic vibrational bands [44]. For the 1% and 2% ZnO nanomodified coating, IR spectra in the spectral range of 1125–750 cm^−1^ looked like the IR spectra of paper, while the characteristic PCL vibrational bands in the spectral range of 1500–1125 cm^−1^ were slightly pronounced, indicating a lower degree of polymerization and higher penetration within the paper structure (Figure 8d,e). 

The photodegradation mechanism of PCL occurred due to the presence of light absorbing species and chromophore functional groups, i.e., carbonyl, resulting in the absorption of UV radiation by carbonyl groups present on the polymer backbone and proceeding via Norrish II-type reaction, which explains the chain scission (decrease in molecular weight), and the formation of C=C double bonds and hydroperoxide O–H at newly formed chain terminals [45]. The exposure of prints coated with 1% TiO_2_ PCL to UV radiation (Figure 8a) caused the increase in vibrational band intensities at 3265 cm^−1^ (O–H vibrational bands), and broadening and movement towards higher wavenumbers. For the prints coated with 2% TiO_2_/PCL (Figure 8b) after UV exposure, the carbonyl band was shifted from 1720 to 1722 cm^−1^. This vibrational band broadened with the increase in time to UV exposure, probably due to the formation of new radicals (C=O) and the overlap with those vibrational bands. The broadening of this band could be attributed to the formation of photodegradation products (C=C) and their mutual overlapping. In addition, the formation on new bands around 1650 cm^−1^ after 18 h of UV exposure indicated the formation of C=C bonds and confirmed the photodegradation mechanism od PCL. The formation of this band was visible for the 3% TiO_2_ nanomodified coating after 18 h of UV exposure (Figure 8c), while in other samples, this could be explained as the broadening of the carbonyl peak and not as the formation of a new band. In addition, with UV exposure, the band at 1462 cm^−1^ was shifted towards 1458 cm^−1^. The vibrational band at 1292 cm^−1^ was shifted to 1294 cm^−1^ and slightly decreased by the influence of UV radiation. All these changes could be attributed to a change in polymer crystallinity resulting from photodegradation, the changes in PCL characteristic functional groups, and the absence of intermolecular interactions. For samples coated with nanomodified PCL/ZnO NPs, UV radiation caused significant changes in the IR spectra (Figure 8d,f). The absorption peak at 1539 cm^−1^ gradually disappeared with the increase in exposure time to UV radiation. The greatest changes occurred in the spectral range of 1250–1125 cm^−1^, where vibrational bands disappeared.

### 3.5. Fluorescence Spectroscopy

**Figure 9:**
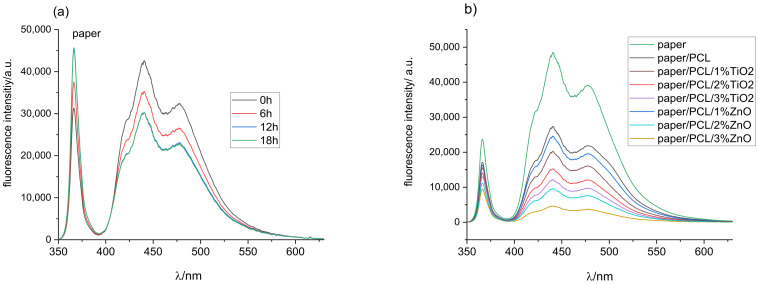
Fluorescence intensity spectra of (**a**) unprinted paper before and after exposure to UV radiation, and (**b**) unprinted paper coated with neat PCL and nanomodified PCL coatings.

Fluorescence occurs when fluorescent molecules absorb the light of the corresponding wavelength to move from the ground to the excited state, followed by the re-emission of energy in the form of visible light when returning to the ground state [46]. One of the advantages of fluorescence spectroscopy measurement is its high sensitivity, and even small concentrations of fluorescent material can be detected. Before examining print fluorescence, it was necessary to address the influence of coating on paper fluorescence and the fluorescence stability of paper substrate exposed to UV radiation for 6, 12, and 18 h (Figure 9a,b).

As expected, the measurements of printing substrate (Figure 9a) showed the highest amount of florescence intensity in the blue part of the spectrum (400–490 nm) due to the use of optical brighteners (OBAs) [33,47]. OBAs were added in the paper during the production process to increase paper whiteness [48]. After the exposure of unprinted paper to UV radiation for 6 h, fluorescence intensity decreased. A similar drop in fluorescence intensity could be seen after 12 h of UV exposure, after which paper fluorescence was not further reduced by prolonged exposure to UV radiation. This was confirmed by the high amount of absorbed excitation light at 365 nm for the unprinted paper, which decreased after its exposure to UV radiation, indicating the degradation of the optical whiteners. 

It is important to determine how the coverage of the substrate with different coatings affects the paper’s fluorescence ability, i.e., a smaller amount of excitation light is available, causing less activation of the optical brighteners incorporated into the paper itself. The coating absorbs a certain amount of incident UV light, decreasing the amount of light accessible for activating optical brighteners in the paper itself. As a result, the fluorescence intensity of paper coated with PCL decreased by 55% compared to the untreated substrate (Figure 9b). The addition of different concentrations of TiO_2_ and ZnO nanoparticles into the PCL matrix further decreased the fluorescence intensity of the paper sample. Increasing the concentration of nanoparticles that strongly absorbe in the spear area in the wavelength of the exciting light affects the reduction in paper fluorescence. Since ZnO NPs used in the study strongly absorbed in the UV part of the spectrum from 200 to 374 nm (Figure 1), increasing its concentration in the PCL coating reduced the amount of incident excitation light, which reached the substrate to generate a fluorescent response. Accordingly, fluorescence intensity was the lowest for the paper coated with PCL coating modified with 3% ZnO (Figure 9b). This also confirmed that the initial colouration of the samples appeared to be darker when samples were coated with ZnO NPs due to the higher absorption rate and consequent lower reflection rate (Figure 1 and Figure S1).

**Figure 10:**
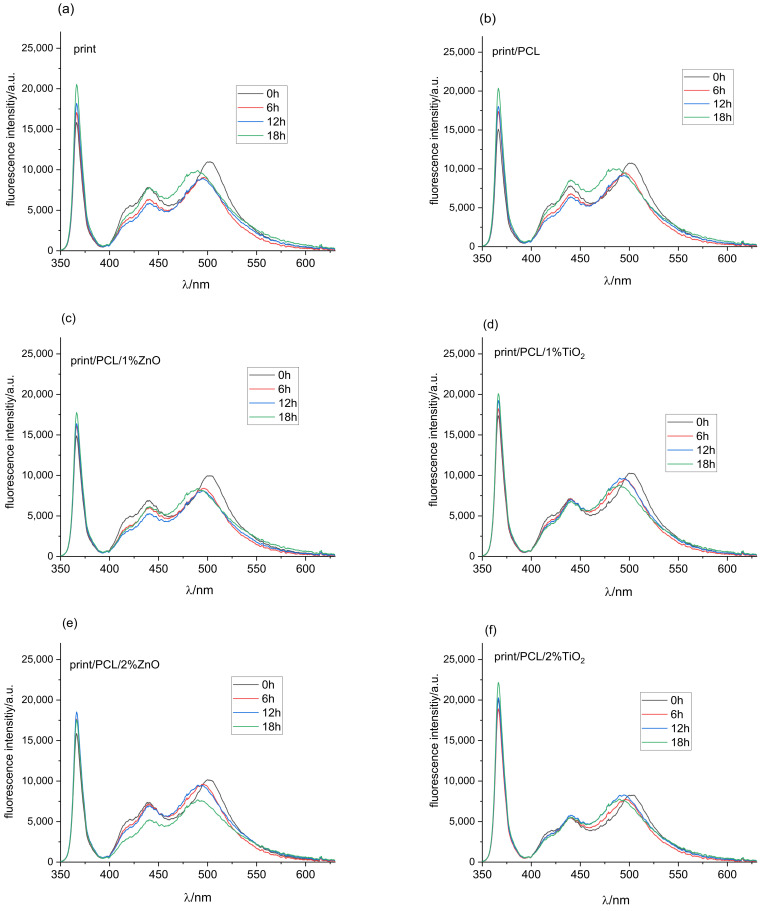

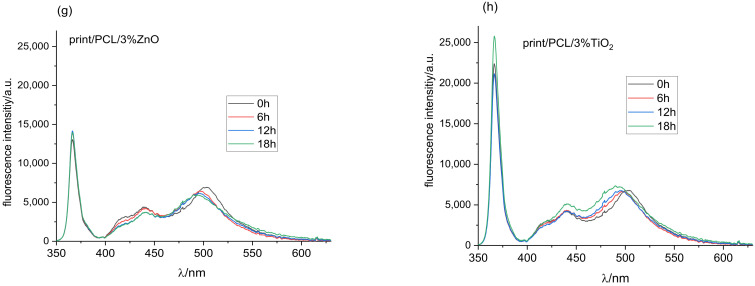
Fluorescence intensity spectra of (**a**) uncoated TC print, (**b**) TC print coated with neat PCL, and nanomodified PCL coatings; (**c**) PCL/1% Zn, (**d**) PCL/1%Ti, (**e**) PCL/2% Zn, (**f**) PCL/2% Ti, (**g**) PCL/3% Zn, (**h**) PCL/3% Ti, before and after 6, 12, and 18 h exposure to UV radiation.

Once the characteristic fluorescence peaks of the substrate had been determined, the fluorescence spectroscopy of the prints could be measured and discussed. Fluorescence intensity spectra of all samples before their exposure to UV radiation followed by 6, 12, and 18 h exposure to UV radiation are shown in Figure 10a. The absorption range at the excitation wavelength of printed samples (Figure 10a) showed that the ratio of absorbed light was much lower than the absorbed intensity of the clear paper substrate (Figure 9a). Due to the absorption, scattering, and re-emission of components within the thermochromic ink, fluorescence intensity spectra were shifted towards higher wavelengths around 510 nm, while the peak at 440 nm corresponded to measurements obtained on the unprinted paper substrates.

As the thermochromic inks used in the study were vegetable-oil-based, their fluorescent response was expected due to their composition of mainly chlorophylls, parinaric acid, and vitamin E [49]. Different oils show different exhibition peaks; sunflower oil exhibits fluorescence peaks in the 430 to 520 nm region [50], extra virgin olive oil between 500 and 720 nm [51,52], rapeseed oil with characteristic spectra from 300 to 500 and from 630 to 730 nm [49]. 

Exposure of the samples to UV radiation for 6 and 12 h (Figure 10a) resulted in a drop in fluorescence intensity and a shift of the peak from 510 to 500 nm. Further exposure degraded the ink, mostly the binder, of which vegetable oil is an integral part, leaving a higher ratio of the paper substrate available for the absorption of UV light, which was confirmed with the increase in fluorescence intensity corresponding the fluorescence spectra of paper substrates (Figure 9a).

Fluorescence intensity spectra of the print coated with PCL (Figure 10b) corresponded to the florescence intensity spectra of the print for unaged samples, and samples exposed to UV radiation for 6 and 12 h. After 18 h of exposure, fluorescence intensity increased in the area from 425 to 450 nm, indicating that PCL to some infinitesimal extent protected the paper even though it was not successful in protecting the print itself.

Increasing TiO_2_ NPs in the PCL solution decreased the absorption of the fluorescence excitation light at 365 nm (Figure 10d,f,h). On the other hand, ZnO particles in the 1% and 2% concentrations did not influence absorption at the given wavelength (Figure 10c,e). The 3% of ZnO particles in the PCL polymer matrix increased absorption in the UV part of the spectrum (Figure 10g). The exposure of samples to UV radiation slightly reduced the absorption of light in the UV part of the spectrum for all examined samples. Comparing the results for ZnO and TiO_2_ nanomodified coatings after 18 h hours of exposure to UV radiation showed that samples coated with 3% TiO_2_ had a 43% higher level of fluorescence intensity in the excitation part of the spectrum. 

The fluorescence response in the visible part of the spectrum showed that the 1% ZnO coatings show similar characteristics as those of the untreated samples after 6 and 12 h of exposure to UV radiation, while fluorescence peaks after 18 h showed much lower intensities (Figure 10c), and the main peaks were not completely shifted towards lower wavelengths, indicating the retention of binder fluorescence. This could similarly be confirmed for 2% ZnO nanomodified coatings, where a decrease in fluorescence intensity and a shift of peaks towards lower wavelengths were observed. Further UV radiation exposure only decreased intensity.

As expected, the 3% ZnO coating showed the lowest fluorescence intensity in the visible part of the spectrum (Figure 10g) since the amount of light absorption increased with the increasing concentration of ZnO nanoparticles. Exposure to UV radiation did not cause any major changes in the intensity and position of the fluorescent peaks.

The visible spectrum of samples coated with the PCL modified with 1% TiO_2_ nanoparticles (Figure 10d) showed higher degradation of the peak around 500 nm, which had been moved towards the fluorescence peaks of OBAs in the paper after 6 h of exposure of the sample to UV radiation. After 12 and 18 h of exposure, fluorescence intensity spectra were almost identical, with an infinitesimal increase in fluorescence intensity in the region between 450 and 475 nm.

The 2% TiO_2_ nanoparticle coating (Figure 10f) showed an identical fluorescence curve response in the visible part of the spectra as that of the 1% TiO_2_ nanomodified coating, with the exception of a difference in fluorescence intensity, namely, it was equal in this case regardless of the exposure of samples to UV radiation.

The last of the examined samples, the TC print coated with 3% TiO_2_, showed an identical fluorescence response in the 400 to 450 nm region of the spectra as that of the untreated and exposed samples for the duration periods of 6 and 12 h, while a clear shift of the broad peak from 500 to 490 nm could be seen (Figure 10h). The sample exposed to 18 h of UV radiation showed slightly greater fluorescence intensity, indicating a further shift towards wavelengths corresponding to the OBAs in the paper.

Taking into account all the presented results, it could be concluded that the overall degradation of coated TC prints was the result of a combined effect due to the presented degradation mechanisms of paper, TC ink binder, neat PCL, and nanoparticles. The degradation of TC prints coated with nanomodified PCL coating by the action of UV radiation could be attributed to photochemical reactions arising from the absorption of UV radiation by light-absorbing groups present on the PCL backbone, and could mainly be expressed by Norrish reactions: photoionization (Norrish I) and chain scission (Norrish II) [45]. This mechanism was clearly presented by the FTIR spectroscopy, with the most notable changes occurring after 18 h of exposure to UV radiation. The main light-absorbing groups present on the PCL backbone and TC printing ink responsible for the initiation of photochemical reactions were carbonyl groups. SEM microscopy (Figure 6) indicated the photo-oxidation of the sample surface, while FTIR spectroscopy indicated changes in the amorphous part of the PCL structure, and changes occurring during cross-linking and chain scission (Figure 8). Fluorescence spectroscopy (Figure 10) confirmed the results of FTIR spectroscopy, the degradation of the carbonyl groups present in vegetable oil (ink binder), and the consequent degradation of the TC ink binder. The degradation of the TC microcapsules’ polymer shell envelope probably occurred due to ink binder degradation, which in the end caused the degradation of a leuco-dye–developer complex inside the TC microcapsules, and the consequent degradation of colour. The degradation of the thermochromic effect by UV radiation was the greatest when the prints had been exposed for 18 h, with total destruction of the TC effect for the uncoated and TC print coated with neat PCL. The addition of NPs in the PCL solution improved the UV stability of the coated TC prints due to the absorption of a certain amount of incident UV radiation, which was clearly confirmed by fluorescence spectroscopy.

## 4. Conclusions

Thermochromic printing inks in the function of smart materials have many application possibilities due to their simple communication with users and their attractiveness, which ultimately add value to the products. However, their limiting circumstance is poor stability against UV radiation, which limits their application. Despite this very well known fact, there is a lack of research related to the development of UV-protective coatings for TC prints. Therefore, the aim of this paper was to investigate the applicability of PCL nanocomposite coating to protect TC prints from UV degradation. Obtained results showed that, despite the higher band gap in comparison to that of TiO_2_, ZnO nanoparticles in a PCL matrix did not improve the UV stability of TC prints in higher proportion than that of TiO_2_. In addition, those results and results from NP UV absorbance determination indicated that all changes within the TC microcapsules and consequent influenced changes in TC print coloration were the result of UV radiation in wavelengths below 329 nm. Additionally, in this research, SEM micrographs showed that UV degradation caused the formation of voids, craters of nanomodified PCL coatings. Moreover, the addition of TiO_2_ nanoparticles into the PCL matrix more effectively promoted the UV degradation of nanomodified PCL coating compared to in ZnO nanoparticles. UV irradiation caused changes in the IR spectrum of the PCL coating, which could be attributed to photodegradation, changes in PCL characteristic functional groups, and the absence of intermolecular interactions. 

This paper proves that the use of fluorescence spectroscopy in the evaluation of UV absorption potential and of the degradation mechanisms of biodegradable polymer paper coatings is a promising tool. In addition, results of fluorescence spectroscopy indicate that the nanomodified PCL coating absorbed a certain amount of incident UV radiation and indicated the degradation of vegetable oil in the printing ink binder. Results indicate that changes occurring in the colouration of the prints could be described by colorimetric measurements and fluorescence spectroscopy, while FTIR spectroscopy only indicated the degradation mechanisms of the TC ink binder and nanomodified coating, but it could not indicate any degradation of the TC microcapsules. However, fluorescence spectroscopy could be used as an auxiliary tool to confirm FTIR spectroscopy results. 

This research showed the possibility of using environmentally friendly nanomodified PCL coating to preserve the thermochromic effect of TC offset prints from the effects of UV radiation, and opens up the possibility of new research in this area. Future research will be focused on the optimization of TiO_2_ weight concentrations in a PCL solution. Moreover, research shows the possibility of expanding applications of TC prints that were limited due to their poor resistance to UV light. For example, TC ink can be used in the development of smart packaging, but this application is limited due to its fast colour degradation when exposed to UV radiation. Nanomodified PCL coatings are a promising material in development and as mechanical protection of different paper-based packaging products. Thus, the combination of TC prints and PCL nanomodified coating is a promising structure in the development of smart packaging with extended applications.

## Figures and Tables

**Table 1:** Composition of PCL nanocomposites (wt %) and designation.

Sample	Ethyl-acetate, %	PCL, %	TiO_2_, %	ZnO, %
P/PCL	90	10	-	-
P/PCL/1Ti	89	10	1	-
P/PCL/2Ti	88	10	2	-
P/PCL/3Ti	87	10	3	-
P/PCL/1Zn	89	10	-	1
P/PCL/2Zn	88	10	-	2
P/PCL/3Zn	87	10	-	3

## Data Availability

Not applicable.

## Supplementary Materials

The following supporting information can be downloaded at https://www.mdpi.com/article/10.3390/polym14071484/s1. Figure S1: CIELAB lightness L* of prepared samples before UV exposure in dependence on temperature at heating (-H) and cooling (-C); Figure S2: FTIR spectra of paper, TC print, and TC print coated with neat PCL coating, and nanomodified PCL coating.
