# Supplementary material for: Improvement in Thermochromic Offset Print UV Stability by Applying PCL Nanocomposite Coatings
Source: Polymers (Basel). 2022 Apr 6;14(7):1484. doi: 10.3390/polym14071484 (PMC9002658; doi:10.3390/polym14071484)
Supplement: Supplementary file 1 [file polymers-14-01484-s001.zip › polymers-1665440-supplementary.pdf]

Supplementary materials

Improvement of thermochromic offset prints UV stability by applying PCL nanocomposite coatings

Marina Vukoje [1]*, Rahela Kulčar [2]*, Katarina Itrić Ivanda [1], Josip Bota[1] and Tomislav Cigula[1]

[1] University of Zagreb Faculty of Graphic Arts, Getaldićeva 2, 10000 Zagreb, Croatia; katarina.itric.ivanda@grf.unizg.hr (K.I.I); josip.bota@grf.unizg.hr (J.B); tomislav.cigula@grf.unizg.hr (T.C)

* Correspondence: marina.vukoje@grf.unizg.hr (M.V); rahela.kulcar@grf.unizg.hr (R.K)

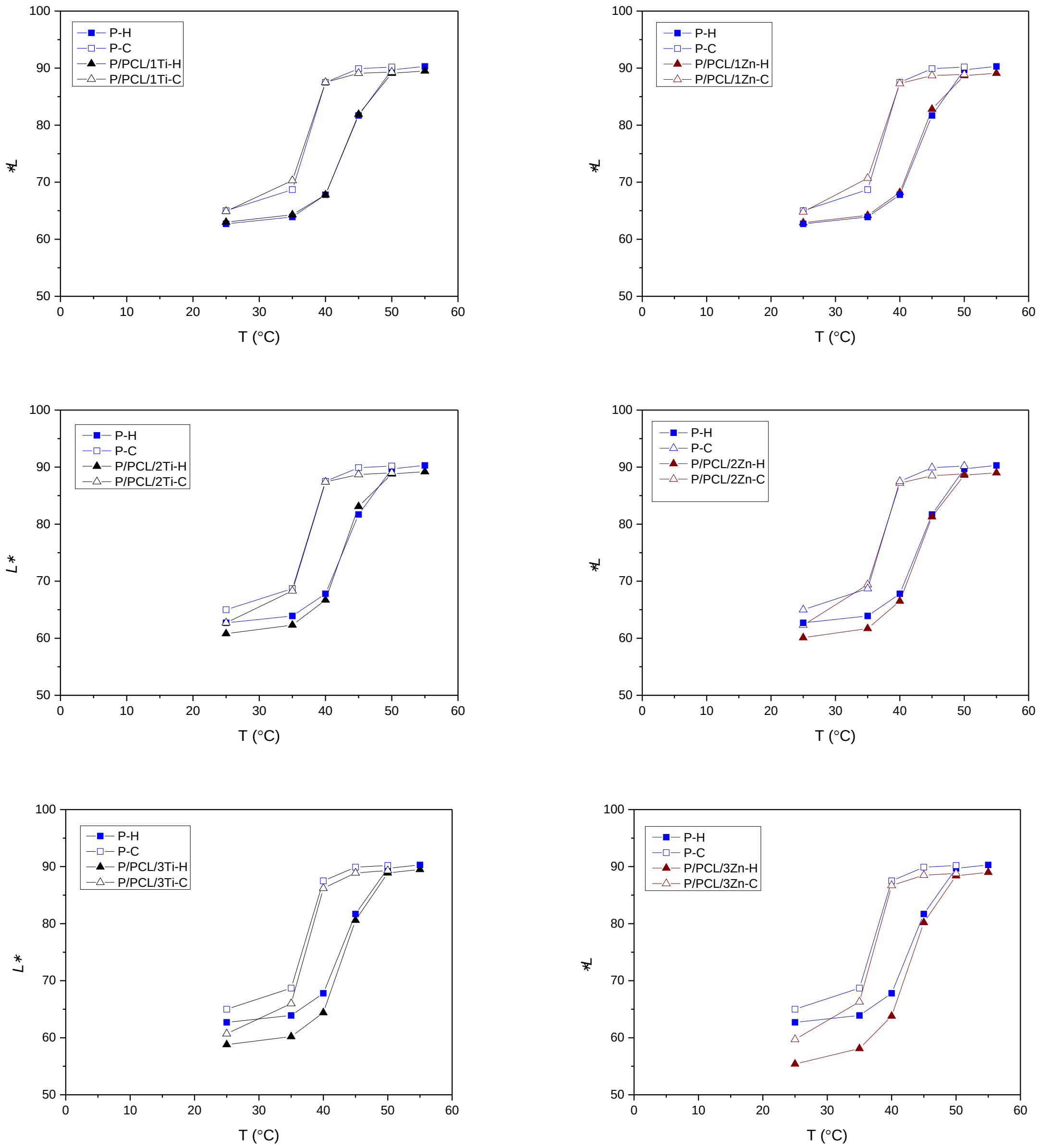

Figure S1. CIELAB lightness *L** of prepared samples before UV exposure in dependence on temperature at heating (-H) and cooling (-C)

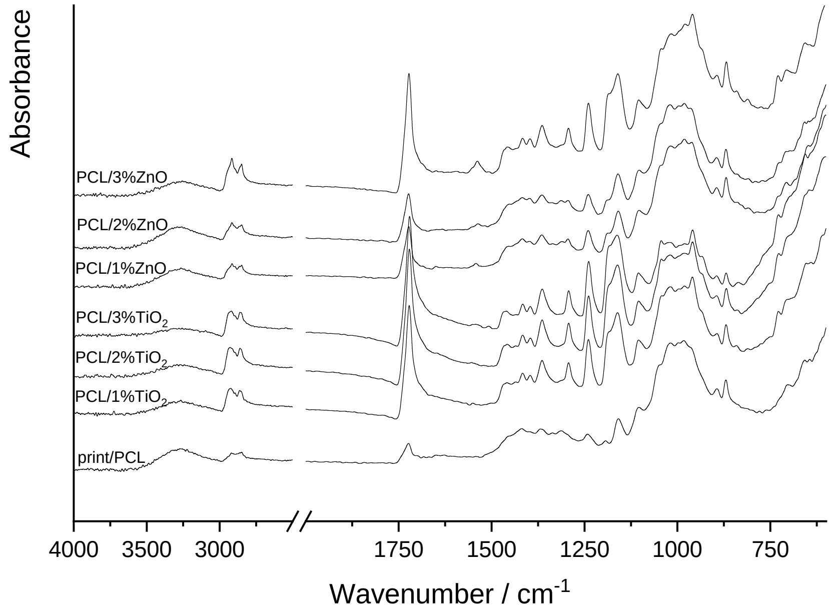

Figure S2. FTIR spectra of paper, TC print and TC print coated with neat PCL coating and nanomodified PCL coatings
